# Proteasome Dysregulation in Parkinson’s Disease: Insights from Blood-Based Analyses

**DOI:** 10.1007/s12035-025-05624-8

**Published:** 2025-12-24

**Authors:** Alfonso Di Costanzo, Carmela Fusco, Rosa Camerlingo, Sara Serafini, Antonio Minò, Cristiana Tiberio, Gianna Palmieri, Carmela Matrone, Ennio Cocca, Antonella Angiolillo

**Affiliations:** 1 Department of Medicine and Health Sciences, Centre for Research and Training in Medicine of Aging, University of Molise, 86100 Campobasso, Italy; 2 Molise Regional Health Service, ASREM, Campobasso, Italy; 3 Institute of Biosciences and BioResources, National Research Council (CNR-IBBR), 80131 Naples, Italy; 4 Cell Biology and Biotherapy Unit, Istituto Nazionale Tumori, IRCCS, Fondazione G. Pascale, 80131 Naples, Italy; 5 Department of Neuroscience, Faculty of Medicine, University of Naples Federico II, 80131 Naples, Italy

**Keywords:** Parkinson’s disease, APEH, PSMB5, Proteasome activity, Peripheral blood biomarkers

## Abstract

Parkinson’s disease (PD) is a neurodegenerative disorder that is characterized by α-synuclein aggregation, mitochondrial dysfunction, and impaired proteostasis. The peripheral biomarkers that reflect these cellular perturbations remain incompletely defined. This study aimed to evaluate the enzymatic activity and gene expression of two key protein degradation enzymes, Acyl PEptide Hydrolase (APEH) and Proteasome Subunit Beta Type-5 (PSMB5), in the peripheral blood of PD patients and to relate these findings to the severity of the disease. Thirteen PD patients and 13 age-matched healthy controls (HLT) were recruited. APEH and PSMB5 chymotrypsin-like (CT-like) activity were measured in whole blood, erythrocytes and immune cell fractions. Gene expression analysis was performed for *APEH* and *PSMB5* and their related genes, plus other genes typical of parkinsonism or indicative of metabolic alterations: N(alpha)-acetyltransferase (*NAA10*) Aminoacylase 1 (*ACY1*), Ubiquitin-activating enzyme 1 (*UBA1*), Ubiquitin conjugating enzyme E2 I (*UBE2I*), Parkin RBR E3 ubiquitin protein ligase (*PRKN*), Alpha-synuclein (*SNCA*), Parkinsonism associated deglycase DJ-1 (*PARK7*), and mitochondrial Mn-SOD (*SOD2*). APEH activity did not differ significantly between PD and HLT in whole blood cells or cellular fractions. Conversely, PSMB5 CT-like activity was reduced in lymphocytes from PD patients, whereas erythrocytes and whole blood exhibited elevated activity. No changes in *APEH* and *PSMB5* expression were observed, while *PARK7* significantly decreased in patients with PD. Correlation analysis showed that proteasomal changes correlated with disease severity, and cognitive impairment. Our findings revealed compartment-specific proteasomal dysregulation in the peripheral blood of PD patients, suggesting systemic proteostasis imbalance. These alterations appeared more pronounced in patients with more severe clinical progression. This study supports the potential of peripheral proteasomal activity profiles as biomarkers linked to PD progression, warranting further investigation in larger cohorts.

## Introduction

Parkinson’s disease (PD) is a progressive neurodegenerative disorder characterized by the selective loss of dopaminergic neurons in the substantia nigra and accumulation of intraneuronal α-synuclein aggregates. The build-up of misfolded proteins such as α-synuclein contributes to proteostatic stress, oxidative damage, and mitochondrial dysfunction, forming a vicious cycle that drives neurodegeneration [1–3].

Owing to its central role in protein clearance, the proteasome is increasingly recognized as a promising therapeutic target in PD, with several small-molecule activators showing efficacy in preclinical models [4–6]. Proteasome activity, particularly the chymotrypsin-like (CT-like) activity mediated by the β5 catalytic subunit encoded by Proteasome Subunit Beta Type-5 (*PSMB5*), has been shown to decline with age and in the context of PD [7–9] and other neurodegenerative diseases [10, 11]. Recent studies have also implicated the immunoproteasome subunit β5i, encoded by Proteasome Subunit Beta Type-8 (*PSMB8*), in PD pathology [12].

Importantly, CT-like activity represents the rate-limiting step in bulk proteolysis and is highly responsive to oxidative and proteotoxic stress [12, 13]. This makes PSMB5 a sensitive and biologically meaningful marker of systemic proteasomal function, reflecting both the constitutive (β5/PSMB5) and inducible (β5i/PSMB8) catalytic sites and thereby capturing potential shifts in proteasome composition under pathological conditions. Impaired proteasomal degradation facilitates the accumulation of toxic protein aggregates, which in turn further suppress proteasome activity, amplifying proteotoxic stress and neuronal injury [14].

Aside from the proteasome, the Acyl PEptide Hydrolase (APEH), also known as oxidized protein hydrolase or acylamino acid–releasing enzyme, is a serine exopeptidase that belongs to the prolyl oligopeptidase (POP) family [15, 16]. APEH cleaves N-terminal acylated amino acids from peptides and proteins, a key function, given that N-terminal acetylation is among the most prevalent co-translational modifications in eukaryotes [17]. Traditionally viewed as a housekeeping enzyme, APEH has been increasingly recognized for its role in degrading oxidatively modified proteins, including amyloid-β peptides, and for participating in broader redox and proteolytic responses [18–20]. Reduced APEH activity has been reported in several oxidative stress–associated disorders, suggesting a potential link between APEH enzymatic regulation and systemic damage response [7, 18, 21].

In this context, parallel investigation of PSMB5 and APEH activity may offer novel insights into the peripheral proteostatic and oxidative stress responses in PD.

## Materials and Methods

### Study Design

The participants in this study were enrolled at the Center for Research and Training in Medicine of Aging at the University of Molise. A total of 26 individuals were recruited, comprising 13 patients diagnosed with PD and 13 healthy controls (HLT). The demographic and clinical characteristics of the study groups are presented in Table 1. The sex distribution reflects the availability of patients recruited during the study period; no intentional selection was applied. All patients underwent comprehensive anamnesis as well as general and neurological examinations. The inclusion criteria for patients with PD required a “clinically established” diagnosis of PD according to the criteria set by the Movement Disorder Society (MDS). Patients with cognitive decline preceding or occurring within less than 2 years of the onset of motor symptoms were excluded from the study.

**Table 1:** Clinical and demographic characteristics of participants. The table summarizes the demographic, clinical, and comorbidity profiles of the PD patients (*n* = 13) and age-matched HLT control subjects (*n* = 13) included in the study. Additional rows show the prevalence of common comorbid conditions and current medication use

	HLT *n* = 13	PD *n* = 13	*p*-value
**Clinical features:**			
Age (years)	68.5$\pm$7.3	70.1$\pm$6.3	ns
BMI at enrolment (kg/m^2^)	24.8$\pm$3.7	26.9$\pm$4.7	ns
Education (years)	15.6$\pm$5.9	10.4$\pm$4.3	0.016
Family history (n. of parents affected)	0/13	0/13	ns
Sex			
Males	9/13	8/13	ns
Females	4/13	5/13	ns
Smokers	7/13	9/13	ns
Alcohol > 4 units/day	0/13	1/13	ns
Time of clinical diagnosis (years)	0	8.4$\pm$6.5	
MMSE	28.3$\pm$1.2	17.2$\pm$7.4	0.0009
ADL	6.0$\pm$0.00	3.8$\pm$2.5	
IADL	8.0$\pm$0.00	3.9$\pm$3.5	
UPDRS		61.0$\pm$42.2	
HOEHN and YAHR		2.7$\pm$1.1	
SCHWALB and ENGLAND		73.8$\pm$32.3	
**Other pathologies:**			
Hypertension	4/13	3/13	ns
Diabetes	1/13	2/13	ns
Dyslipidaemia	3/13	6/13	ns
Carotid atheromasia, aneurysm	0/13	0/13	ns
Tia/stroke	1/13	0/13	ns
Other neurodegenerative disorders	0/13	0/13	ns
Autoimmune disorders	0/13	0/13	ns
Cirrhosis, steatosis, gallstones	1/13	0/13	ns
Current or past tumoral diseases	1/13	1/13	
**Medications:**			
Antihypertensives	4/13	2/13	ns
Hypoglycemics	1/13	2/13	ns
Hypolipidemic or lipid-lowering agents	2/13	3/13	ns
L-Dopa	0/13	13/13	< 0.0001

Data are expressed as the mean ± standard deviation or the number of affected individuals over the total group size. Statistical comparisons were conducted using unpaired *t*-tests or chi-squared tests, where appropriate. Significant differences (*p* < 0.05) are indicated. “ns” denotes non-significant differences

The Mini-Mental State Examination (MMSE) was used to evaluate global cognition, Basic Activities of Daily Living (BADL), and Instrumental Activities of Daily Living (IADL) were used to evaluate functional abilities and the Cumulative Illness Rating Scale (CIRS) to assess patients’ comorbidities.

The PD patients included in our study were under dopaminergic treatment with L-dopa as part of their regular therapeutic regimen.

The study adhered to the ethical principles outlined in the Declaration of Helsinki and complied with the approved national and international guidelines for human research.

The Institutional Review Board (IRB) of the University of Molise reviewed and approved this study (IRB Prot. n. 17/2020). Written informed consent was obtained from all the participants.

### Collection of Blood Samples

Blood samples were collected between 8:00 and 8:30 a.m. following an overnight fasting period of at least 8–10 h. Antecubital venous blood was drawn into vacutainer tubes (Becton & Dickinson, Milan, Italy), including one tube with a separating gel and a clot activator for serum collection, one sodium citrate tube for plasma collection, two lithium heparin tubes for the isolation of blood cell fractions, and one EDTA tube for DNA and RNA extraction. Serum and plasma were obtained by centrifugation at 1500 rcf for 10 min using an Eppendorf® 5810 R centrifuge. All samples were subsequently stored at − 80 °C until further processing.

### Sorting/Flow Cytometry

To isolate the peripheral blood leukocyte (PBL) fraction, 2 mL of whole blood was lysed using the BD Pharm Lyse™ lysing solution (AB_2869057; Becton & Dickinson, Milan), following the manufacturer’s guidelines. Each cell sub-population was sorted via Fluorescence-Activated Cell Sorting (FACS) based on “side scatter” and “forward scatter” parameters using the FACS ARIAIII system and analyzed with Diva 8.0 software (SCR_001456; Becton & Dickinson, Franklin Lakes, NJ, USA).

To confirm the separation and purity of the cell populations, post-sorting staining was conducted using mouse anti-human CD3 PE-Cy5 (AB_395741; BD Pharmingen, Milan, IT) for lymphocytes, mouse anti-human CD14 PE-VIO770 (AB_2660180; Miltenyi Biotech, Bologna, IT) for monocytes, and mouse anti-human CD15 PE (AB_11154049; BD Pharmingen) for granulocytes. All the antibodies were applied at a concentration of 2 µg/mL for 30 min at 4 °C. Blood cell fractions were stored at − 80 °C until further processing.

### RBCs Isolation

Red blood cells were isolated using Ficoll® Paque Plus from GE Healthcare (DBA, Milan, IT), employing a method involving a rapid centrifugation step. A 1 mL aliquot of whole blood, diluted 1:2 with 1X D-PBS buffer (Sigma-Aldrich, Milan, IT), was carefully layered atop 1.5 mL of Ficoll® Paque Plus, which was positioned at the bottom of a centrifuge tube. This sample was then centrifuged using an Eppendorf® 5810 R with a swinging arm rotor (S-4–104) at a speed of 400 rcf, with an acceleration setting of 2 and deceleration setting of 2, for 30 min at 25 °C. Following this procedure, the distinct cellular components of the blood, visible as well-defined layers, were gently removed, leaving erythrocytes at the bottom of the tube. They were subsequently stored at − 80 °C for future processing.

### Total Protein Extraction from Blood Cells

Whole blood and blood cell fraction samples stored at − 80 °C were gradually thawed and subsequently resuspended in an appropriate volume of extraction buffer (100 mM HEPES, 50 mM NaCl, 1 mM PMSF, and 0.5% NP-40). The samples were vortexed for 1 min and placed on ice for 30 min, vortexing at 10-min intervals. The samples were then centrifuged at 11,000  rcf for 45 min to isolate the supernatant containing the soluble protein fraction. To the supernatant, 5% glycerol was added for cold storage. Total protein concentration was quantified using the Bradford assay.

### APEH Exopeptidase Assay

The amino-peptidase activity of APEH was quantitatively assessed by spectrofluorometry, employing the fluorogenic substrate acetyl-Met-7-amido-4-methylcoumarin sourced from Bachem (Bubendorf, Switzerland). The release of the fluorescent product, 7-amino-4-methylcoumarin, was monitored using a Shimadzu RF-6000 spectrofluorometer with excitation and emission wavelengths of 380 nm and 460 nm, respectively. Reaction mixtures, each with a volume of 1 mL, contained specified amounts of protein extract (140 µg) in 50 mM Tris–HCl at pH 7.5, and were pre-incubated at 37 °C for 3 min. Subsequently, the substrate was introduced at a final concentration of 0.5 mM, and the release of the product was measured. Enzymatic activity was calculated based on the initial linear phase of product release. Enzymatic activity was expressed in arbitrary units (U), where one unit (μmol/min) was defined as the amount of enzyme that catalyzed the conversion of one micromole of substrate per minute under the specified assay conditions. All experiments were conducted in triplicates using two distinct protein preparations.

### Proteasome 20S Subunit Beta 5 Chymotrypsin (CT)-Like Assays

The synthetic fluorescent substrate N-succinyl-Leu-Leu-Val-Tyr-7-amido-4-methylcoumarin (Suc-LLVY-AMC) (Sigma-Aldrich, Milan, IT) was employed at a final concentration of 0.080 mM to assess the CT-like activity of the PSMB5 within the protein extract. The reaction mixture, with a total volume of 0.9 mL, contained the specified quantities of protein extract (140 µg) and was preincubated in 50 mM Tris–HCl at pH 7.5. Subsequently, Suc-LLVY-AMC was introduced, and the release of the fluorescent product, 7-AMC, was monitored over a 10-min period using a Shimadzu RF-6000 spectrofluorometer. The excitation and emission wavelengths were 380 and 460 nm, respectively.

All experiments were conducted in triplicates using two distinct protein preparations.

### Purification of Total RNA and Quantitative Real-Time PCR (RT-PCR) Analysis

Total RNA was extracted from whole blood samples using TRIzol® LS Reagent (Ambion/Thermo Fisher Scientific, Milan, IT), which is specifically designed for liquid sample processing, following the protocol outlined in the user guide. RNA concentration was measured using a Qubit Fluorometer (SCR_020553; Invitrogen/Thermo Fisher Scientific, USA). Subsequently, the RNA was reverse-transcribed using SuperScript® VILO™ Master Mix (Invitrogen/Thermo Fisher Scientific, Milan, IT). A dilution series of the resulting cDNA served as a template for quantitative real-time PCR amplification to assess the primer efficiency. These assays were conducted on a CFX96 REAL-TIME SYSTEM® (SCR_018064; Bio-Rad, Milan, IT) utilizing 300 nM gene-specific primers and iTaq™ Universal SYBR® Green Supermix (Bio-Rad, Milan, IT) under the following PCR conditions: an initial cycle at 95 °C for 10 min, followed by 40 cycles at 95 °C for 15 s, 60 °C for 30 s, and 72 °C for 30 s. The expression level of β-actin was used as the internal control for normalization. The raw cycle threshold values (Ct values) for the target genes were compared with the Ct value of the β-actin gene (reference gene). All data are expressed as mean expression levels from triplicate experiments, and the final graphical data were derived using the equation:

$$R=({{E}_{target})}^{\Delta Ct\_target(control-sample)}/ {({E}_{ref})}^{\Delta Ct\_ref(control-sample)}$$

Primers were designed using the “Assay design for Universal Probe Library (discontinued service)” (https://primers.neoformit.com/). The primers used are listed in Fig. 3.

### Statistical Analysis

Descriptive statistics, including means and standard deviations, were calculated using GraphPad Prism (version 10.6.1; SCR_002798). Group comparisons for categorical variables were performed using Fisher’s exact test or chi-square test, as appropriate. For statistical analysis, multiple unpaired *t*-tests was used as indicated in the corresponding figure and table legends. Spearman’s correlation analysis was used to analyze the possible links between proteasomal activity and disease severity. We did not perform an a priori power calculation because this investigation was designed as an exploratory, hypothesis-generating study.

## Results

### Clinical Profile of the Subjects Enrolled in the Study

All participants (13 HLT and 13 PD patients) were Caucasian and had completed at least 10 years of education. All patients were considered to have sporadic Parkinson’s disease given the lack of a family history. Nonetheless, in the absence of genetic testing, an underlying genetic contribution cannot be definitively ruled out.

Comprehensive data on age, body mass index (BMI), and key risk factors, including smoking status, alcohol consumption, and comorbid conditions, were collected and are presented in Table 1. Except for the education and MMSE scores, no significant differences were observed between the groups with respect to other risk factors or comorbidities.

Given the extensively studied relationship between cigarette smoking and Parkinson’s disease [22], we performed an analysis comparing smokers and non-smokers. The results demonstrated significantly reduced proteasome activity in monocytes from patient smokers (*F* = 9.7; *p* = 0.01), whereas no significant differences were observed in HLT (*F* ≤ 2.8; *p* ≤ 0.12).

All the patients were treated with L-dopa.

### APEH and PSMB5 (CT-like) Enzymatic Activity and mRNA Levels in Whole Blood and in Cellular Subsets

**Fig. 1:**
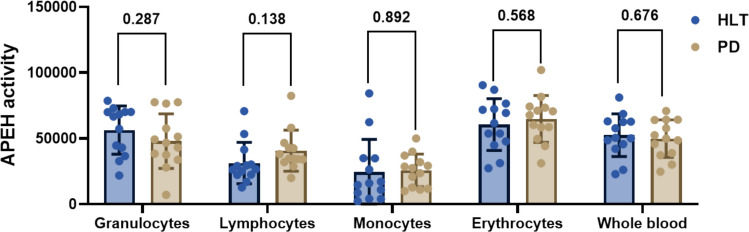
APEH enzymatic activity in leukocytes, erythrocytes and whole blood, from PD patients and HLT controls. Enzymatic activity was quantified in arbitrary units, where one U (μmol/mg) represents the amount of enzyme catalyzing the conversion of one micromole of substrate per minute. Each experiment was conducted on two distinct protein preparations in triplicate. No significant difference was observed between groups (*p* < 0.05, unpaired *t*-test)

We initially evaluated APEH enzymatic activity in whole blood samples obtained from both HLT and PD patients and in their cellular subsets, erythrocytes and leukocytes. As illustrated in Fig. 1, there was no significant difference in APEH activity between the PD and HLT groups. *APEH* mRNA expression levels also revealed no significant difference between the groups (Fig. 3).

**Fig. 2:**
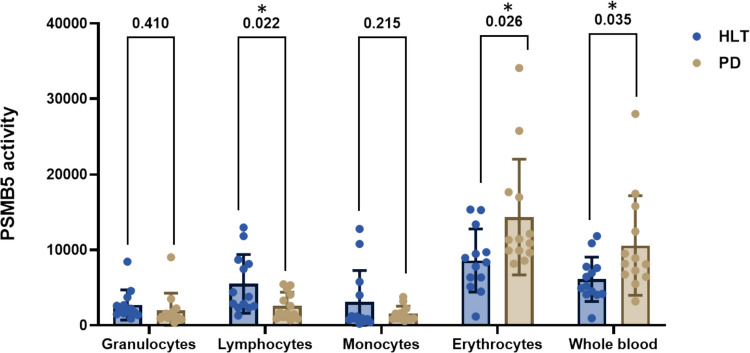
PSMB5 CT-like enzymatic activity in leukocytes, erythrocytes and whole blood from PD patients and HLT controls. Enzymatic activity was quantified in arbitrary units, where one U (μmol/min) represents the amount of enzyme catalyzing the conversion of one micromole of substrate per minute. Each experiment was conducted on two distinct protein preparations in triplicate. The activity was increased in erythrocyte and whole blood of the PD group compared to HLT group, while lymphocytes showed a reduction (*p < 0.05, unpaired *t*-test)

**Fig. 3:**
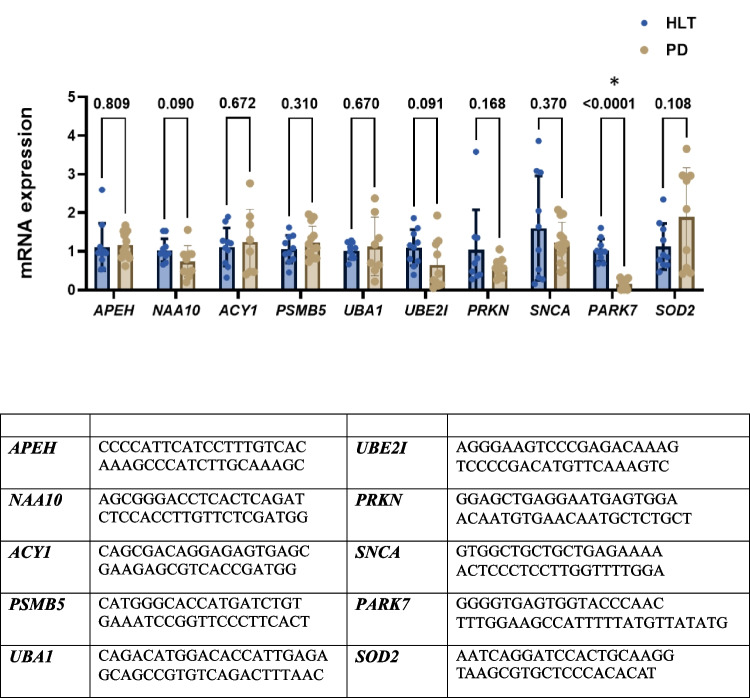
Relative mRNA expression levels of target genes assessed in whole blood samples from HLT and PD patients. Values are presented as the mean $\pm$ standard deviation of relative expression levels, normalized to $\beta$-actin. Gene expression was measured using RT-PCR. The primer sequences used for mRNA analysis are listed in the Table. No significant differences were observed between the groups, except for the *PARK7* gene (* *p* < 0.05, unpaired *t*-test)

We subsequently evaluated the CT-like PSMB5 activity within the same cohort of samples. As illustrated in Fig. 2, the activity was increased in erythrocyte and whole blood and decreased in lymphocytes of the PD compared to HLT group, indicating an alteration of proteasomal function in the peripheral blood cells of PD patients (Fig. 2). However, this alteration in enzymatic activity was not associated with a significant change in *PSMB5* mRNA expression levels (Fig. 3). This suggests the possibility of post-translational regulation of the enzyme in PD patients.

Interestingly, Spearman correlation analysis indicated that lower MMSE scores and higher UDPR scores were associated with reduced PSMB5 CT-like enzymatic activity in lymphocytes (rs = 0.4, *p* = 0.04; and rs =  − 0.4, *p* = 0.04, respectively), while higher UDPR levels correlated with increased PSMB5 CT-like activity in whole blood (rs = 0.4, *p* = 0.04). This opposite correlation pattern across compartments may indicate distinct regulatory or compensatory mechanisms of proteasomal function in response to disease progression.

### Expression Analysis for APEH- and PSMB5-Related Genes, and for Genes Typical of Parkinsonism or Indicative of Metabolic Alterations

We also analyzed the mRNA levels of two metabolically APEH-related enzymes, N(alpha)-acetyltransferase *(*NAA10) and Aminoacylase 1 (ACY1), which are involved in N-terminal acetylation and downstream processing of acetylated amino acids [23, 24]. Like *APEH*, the *NAA10* and *ACY1* genes did not exhibit differential expression between PD and HLT, although a trend towards reduction in PD was observed for *NAA10* (Fig. 3).

Regarding *PSMB5*-related genes, and consistent with a systemic proteostatic dysregulation, we observed a trend toward decreased mRNA levels in blood cells from PD patients for Ubiquitin-conjugating enzyme E2 I (*UBE2I*) and Parkin RBR E3 ubiquitin protein ligase (*PRKN*). In contrast, the expression of the other proteostasis-related gene, Ubiquitin-activating enzyme 1 (*UBA1*), did not differ significantly between PD patients and healthy controls (Fig. 3).

UBE2I (also known as UBC9) mediates SUMO conjugation, a post-translational modification that can target proteins for proteasomal degradation through SUMO-targeted ubiquitin ligases (STUbLs) such as RNF4 [25], whereas PRKN is an E3 ubiquitin ligase that polyubiquitinates substrate proteins, marking them for degradation by the 26S proteasome [26].

UBA1 occupies the apex of the ubiquitin–proteasome cascade, catalysing the activation of ubiquitin and thereby enabling substrate recognition and degradation via the 26S proteasome [27]. Alterations in UBA1 function have been implicated in neurodegenerative processes through impaired proteostasis [28].

Moreover, we found a significant decrease of mRNA levels in PD group for Parkinsonism associated deglycase DJ-1 (*PARK7*) which protects neurons against oxidative stress and cell death [29], together with a trend of decrease for α-synuclein (*SNCA*) mRNA levels (Fig. 3).

Finally, we also found a trend of increase in the expression of mitochondrial Mn-SOD (*SOD2*) (Fig. 3), whose expression levels have previously been reported to be altered in PD [30] as well as in other neurodegenerative diseases [31].

## Discussion

This study presents novel evidence on the peripheral enzymatic landscape of PD, focusing on two proteostasis-related enzymes: APEH and the PSMB5.

To our knowledge, this is the first investigation of exopeptidase APEH in peripheral blood from individuals with PD. Prior work in Alzheimer’s disease (AD) reported a marked reduction of APEH activity in whole blood versus HLT [11]; by contrast, in our PD cohort APEH activity did not differ from HLT. Consistent with this, neither *APEH* mRNA nor two metabolically related genes, *NAA10* and *ACY1*, showed changes between PD and HLT, thus suggesting that, despite shared neurodegenerative features, peripheral APEH dysregulation is not a common biomarker across PD and AD and is unlikely to be centrally involved in peripheral proteostatic dysfunction in PD. Specifically, the *NAA10* gene encodes the catalytic subunit of the NatA complex, the primary N-terminal acetyltransferase responsible for the co-translational modification of proteins [23, 32]. This modification can be recognized and processed by APEH, which removes the N-acetylated amino acids. In contrast, *ACY1* encodes a cytosolic, homodimeric, zinc-binding enzyme responsible for catalyzing the hydrolysis of N-acylated amino acids into amino acids and an acyl group. This gene has been postulated to play a role in catabolism and recovery of acylated amino acids by acting synergistically with APEH to maintain protein homeostasis [24]. Specifically, ACY1 deacetylates N-acetylated amino acids generated by APEH, making it available for reuse.

Proteasomal CT-like activity, largely mediated by the β5 subunit encoded by *PSMB5*, exhibited a more complex, compartment-specific profile. Although multiple post-mortem and experimental studies have shown reduced expression of 20S proteasome subunits in the substantia nigra and other brain regions in PD [10], few studies have examined proteasomal activity in peripheral blood [33]. We prioritized CT-like activity because it represents the rate-limiting step in bulk proteolysis [34] and is highly responsive to proteotoxic/oxidative stress [35]. While our assays did not quantify PSMB8 (β5i) specifically, CT-like readouts integrate catalysis at β5/β5i sites [13, 36]; future β5i-selective measurements will help dissect the contribution of immunoproteasome induction in PD.

The reduced proteasomal activity in monocytes from patient smokers, suggest that in individuals with PD who smoke, the convergence of disease-related ubiquitin–proteasome (UPS) dysfunction and smoke-induced oxidative burden may further compromise proteasome efficiency, potentially intensifying peripheral monocytic activation and systemic inflammation [37, 38]. In effect, cigarette smoking, although consistently linked epidemiologically to a lower incidence of PD, exerts substantial oxidative stress and has been shown to impair proteasome activity in immune cells through the accumulation of oxidized proteins and direct interference with proteasomal complexes [39, 40]. While dedicated studies on proteasomal activity specifically in smoking PD patients remain limited, the existing evidence indicates a plausible additive or synergistic vulnerability of the UPS in this subgroup, warranting targeted investigation.

Recent work shows that the 20S proteasome can degrade intrinsically disordered proteins such as α-synuclein via ubiquitin-independent mechanisms [41], particularly under oxidative stress and aging conditions [42], advising that impairment of the UPS system in PD may increase reliance on alternative degradation pathways, which, if insufficient, could contribute to α-synuclein accumulation. Interestingly, these variations in proteasomal activity were not mirrored by changes in *PSMB5* mRNA, suggesting that transcriptional regulation is not the primary driver.

Notably, the ubiquitin–proteasome system may also influence the autophagy–lysosome pathway, within which Glucocerebrosidase (GBA) functions [43]. Dysfunction of the proteasome can exacerbate the demands on lysosomal degradation, and vice versa, establishing a reciprocal relationship among α-synuclein, GBA activity, and proteostasis. This relationship reflects alterations in multiple pathways rather than isolated gene expression. Consequently, future research should incorporate direct assessments of proteasomal function, lysosomal/autophagic flux, and GBA enzymatic activity to elucidate how these interconnected systems contribute to the observed phenotype.

Previous studies have reported reduced proteasome activity in peripheral blood mononuclear cells (PBMCs) from individuals with PD [7, 44, 45], as well as decreased CT-like activity in lymphocytes from patients treated with L-dopa, consistent with experimental models showing that dopamine can inhibit proteasome function [46, 47]. However, the impact of L-dopa on proteasome activity remains debated, with some studies describing restorative, stimulatory, or compensatory effects [2, 4, 46]. In our cohort, all PD patients were receiving stable L-dopa therapy, a limitation we acknowledge, as it may have contributed to or predisposed individuals to the alterations observed. This consideration is clinically relevant, given that L-dopa is the primary therapeutic approach for most patients, highlighting the need for replication in drug-naïve or longitudinally followed cohorts to distinguish disease-related effects from treatment-related ones.

Of note, erythrocytes and whole blood displayed elevated CT-like activity. This apparent discrepancy may reflect cell-type-specific regulation: erythrocytes, which lack nuclei and rely on post-transcriptional control, exhibit distinct proteasome dynamics compared with nucleated cells. Increased oxidative stress in PD may activate proteasomal function in red blood cells as a compensatory response to remove oxidatively damaged proteins, as reported in other redox-imbalance models [42]. A compensatory up-regulation in erythrocytes could also arise in response to impaired proteostasis in lymphocytes, representing a systemic adaptation to proteotoxic stress [7]. In parallel, we found that the mRNA levels of *PARK7* (DJ-1) were reduced in PBMC from patients with PD. DJ-1 acts as a redox-sensitive molecular chaperone [48] and it has been shown to interact with the 20S proteasome in red blood cells of PD patients [49], consistent with activation of redox-sensitive, ubiquitin-independent degradation pathways. Within this framework, reduced *PARK7* expression in whole blood may limit DJ-1–mediated antioxidant support, thereby exacerbating oxidative/proteotoxic burden and secondarily promoting a compensatory increase in proteasomal turnover, particularly in erythrocytes, the most abundant and oxidation-prone blood cell population. Thus, the elevated CT-like activity observed in whole blood might reflect an adaptive, stress-driven response.

Circulating proteasome alterations have been associated with systemic conditions, including cardiovascular diseases, which often coexist with neurodegenerative disorders and may influence peripheral proteostasis [50]. Recent evidence further supports the interconnection between proteasomal function, mitochondrial homeostasis, and disease mechanisms [51]. In a previous study, we profiled the plasma lipid composition of a cohort of patients with PD [52]. Among the significantly dysregulated metabolites validated through orthogonal projections to latent structures (OPLS), several displayed strong biological relevance to mitochondrial and proteolytic homeostasis (Table 2).

**Table 2:** OPLS-validated plasma metabolites differentially expressed in PD patients and HLT [52]

Metabolite	Type	Direction (c value)	Validated by opls	Biological relevance
Dodecenoyl acylcarnitine	Acylcarnitine	↓ (0.5*)	Yes	Mitochondrial β-oxidation dysfunction
Tetradecenoyl acylcarnitine	Acylcarnitine	↓ (0.8*)	Yes	Incomplete mitochondrial fatty acid oxidation
Hexadecenoyl acylcarnitine	Acylcarnitine	↓ (0.8*)	Yes	Reduced mitochondrial acyl-CoA flux
Octadecenoyl acylcarnitine	Acylcarnitine	↓ (0.8*)	Yes	Mitochondrial lipid metabolism alteration
Methionine-sulfoxide	Oxidized amino acid	↓ (0.8*)	Yes	Protein oxidation marker; reflects proteasomal stress
Ceramide d18:1/24:1	Ceramide	↑ (1.3*)	Yes	Known inhibitor of proteasome CT-like activity (PSMB5)
Docosahexaenoic acid (dha)	Fatty acid	↓ (0.7*)	Yes	Mitochondria–proteasome axis impairment
Arachidonic acid	Fatty acid	↓ (0.6*)	Yes	Inflammation and proteasome regulation
Myristic acid	Fatty acid	↓ (0.3*)	Yes	Energy metabolism and mitochondrial efficiency

Asterisks (*) indicate metabolites validated through orthogonal projections to latent structures (OPLS) analysis. *C* values represent the ratio between mean metabolite levels in PD patients versus healthy controls

In fact, patients with PD showed a consistent reduction in long-chain acylcarnitines (including dodecenoyl-, tetradecenoyl-, hexadecenoyl-, and octadecenoyl-carnitines) and key fatty acids such as arachidonic acid, myristic acid, and docosahexaenoic acid (DHA). These molecules are critical intermediates in mitochondrial β-oxidation and lipid signaling, and their depletion suggests suppression of mitochondrial energy metabolism and metabolic flexibility in PD. Similar reductions in acylcarnitines or their derivatives have been observed in other studies of neurodegeneration [53, 54], including AD [55], with notable differences between female and male patients [56].

In parallel, the levels of ceramide d18:1/24:1, a bioactive sphingolipid, were elevated in patients with PD. Ceramides are known to accumulate under cellular stress [57] and have been shown to inhibit CT-like activity [58] via direct interaction with PSMB5 [59]. These lipid species have also been implicated in the promotion of mitochondrial dysfunction, oxidative stress, and apoptotic signaling [60–62]. Therefore, the observed ceramide elevation may reflect a state of maladaptive lipid signaling that reinforces both proteasome inhibition and mitochondrial stress. In parallel, we observed a significant decrease in methionine-sulfoxide, a known marker of protein oxidation and redox stress, potentially reflecting impaired oxidative protein clearance and an increased burden on the proteasome system [62].

Collectively, these results support a model in which proteasome and mitochondrial dysfunction are tightly linked in PD with evidence of both systemic and cell-specific dysregulation.

## Conclusion

Our findings provide new perspectives on the peripheral enzymatic alterations associated with PD, focusing on the proteostasis-related enzymes APEH and PSMB5.

While APEH activity and expression remained unchanged, PSMB5 CT-like activity showed a compartment-specific pattern, being reduced in lymphocytes but increased in erythrocytes and whole blood. These results suggest distinct, cell-type–dependent regulatory mechanisms likely related to oxidative stress and compensatory proteostatic responses.

Gene expression data from additional proteostasis-related genes, appear to support the biological relevance of the observed enzymatic changes and their connection with mitochondrial and redox pathways.

The distinct proteasomal activity profiles observed across different blood compartments highlight the need for fraction-specific analyses in future biomarker studies.

Some weaknesses of our study include the relatively small sample size and the lack of a priori power calculation, which may have affected the statistical robustness and generalizability of the findings. In addition, the lack of correlation analyses linking APEH and PSMB5 activities with pathological proteins or molecular pathways previously implicated in PD pathogenesis—such as α-synuclein accumulation, mitochondrial dysfunction, or oxidative stress—limits the interpretability of the observed alterations.

Thus, while our data highlight altered proteasomal activity in PD, the results should be considered exploratory. Further investigations in larger, adequately powered, longitudinal cohorts—integrating enzymatic, molecular, and functional assays—will be essential to validate these findings and clarify how peripheral proteostatic alterations relate to established disease mechanisms.

## Data Availability

The data supporting the findings of this study are available upon request from the corresponding author.
